# Identification of candidate cardiomyopathy modifier genes through genome sequencing and RNA profiling

**DOI:** 10.3389/fcvm.2025.1546493

**Published:** 2025-07-28

**Authors:** Malene E. Lindholm, Sarah Abramowitz, Daryl M. Waggott, Megan E. Grove, Frederick E. Dewey, Cuiping Pan, Aleksandra Pavlovic, Ching Shang, Yong Huang, Leore Bensabath, Rachel L. Goldfeder, Pablo Cordero, Ayca Erbilgin, James R. Priest, Hassan Chaib, Megan J. Puckelwartz, Sharlene M. Day, Elizabeth M. McNally, Thomas Cappola, Gerald W. Dorn, Euan A. Ashley, Matthew T. Wheeler

**Affiliations:** ^1^ Stanford Division of Cardiovascular Medicine, Stanford University School of Medicine, Stanford, CA, United States; ^2^ Donald and Barbara Zucker School of Medicine at Hofstra/Northwell, Hofstra University, Hempstead, NY, United States; ^3^ Stanford Clinical Genomics Program, Stanford University School of Medicine, Stanford, CA, United States; ^4^ Forsite Labs, San Francisco, CA, United States; ^5^ Department of Genetics, Stanford University School of Medicine, Stanford, CA, United States; ^6^ Center for Undiagnosed Diseases at Stanford, Stanford University School of Medicine, Stanford, CA, United States; ^7^ Program in Biomedical Informatics, Stanford University School of Medicine, Stanford, CA, United States; ^8^ Department of Medicine, University of California, Los Angeles, CA, United States; ^9^ Tenaya Therapeutics, San Francisco, CA, United States; ^10^ Center for Genetic Medicine, Department of Medicine, Northwestern University Feinberg School of Medicine, Chicago, IL, United States; ^11^ Division of Cardiovascular Medicine, Department of Medicine, University of Pennsylvania, Philadelphia, PA, United States; ^12^ Center for Pharmacogenomics, Department of Internal Medicine, Washington University School of Medicine, St. Louis, MO, United States

**Keywords:** hypertrophic cardiomyopathy, *MYH7*, modifier, genome sequencing, left ventricular hypertrophy

## Abstract

**Background:**

Phenotypic heterogeneity is apparent among individuals with putative monogenic disease, such as familial hypertrophic cardiomyopathy. Genome sequencing (GS) allows interrogation of the full spectrum of inborn genetic variation in an individual and RNA profiling provides a snapshot of the cardiac-specific pathogenic effects on gene expression.

**Objectives:**

Identify candidate genetic modifiers of hypertrophic cardiomyopathy phenotype.

**Methods:**

We performed GS of 48 individuals with variants in *MYH7*, the gene encoding beta myosin heavy chain, and a personal or family history of cardiomyopathy. The genome sequences were annotated with a custom pipeline optimized for cardiovascular gene variant detection. We utilized multiple lines of evidence to prioritize genes together with rare variant gene-based association testing to identify candidate genetic modifiers.

**Results:**

GS identified the *MYH7* variant in all 48 cases. Several variants were reclassified based on best available data. We identified known disease-associated genes (*MYBPC3, FHOD3*), *a priori* candidate modifiers (*ATP1A2*, *RYR2),* and novel candidate modifiers of cardiomyopathy including *PACSIN3* and *SORBS2*. We identified regulatory variants and intergenic regions associated with the phenotypes. Using RNA profiling, we show that several genes identified through gene-based association testing are differentially regulated in human hypertrophic cardiomyopathy, and in models of disease.

**Conclusion:**

Evaluation of the whole genome, even in the case of alleged monogenic disease, leads to important new insights. The identified variants, regions, and genes are candidates to modify disease presentation in cardiomyopathy.

## Introduction

Familial cardiomyopathy is a major cause of morbidity and mortality. Hypertrophic cardiomyopathy (HCM) was first mapped by positional cloning to the cardiac beta myosin heavy chain (*MYH7*) (1, 2). Subsequently variants in other sarcomeric genes were found to be pathogenic for HCM. Today, a pathogenic genetic variant is identified in <50% of HCM cases (3), where rare and private variants in myosin binding protein C (*MYBPC3*) and *MYH7* are most common (4). High variability in disease presentation, penetrance and expressivity has caused some to question the single variant nature of these diseases and led to speculation that additional genetic variation may be contributing (5–7). Disease heterogeneity, both among families with identical “disease-causative” variants and between individuals within these families, can span severity and presentation and complicates diagnosis and prognosis (6). Importantly, polygenic risk can explain some variability in disease presentation in patients with HCM (8). Additionally, several groups demonstrated that patients harboring multiple rare variants in disease-associated genes may develop severe phenotype or present earlier (9–11). Genome-wide association studies have identified large effect variants with moderate population frequencies (8, 12), while small to moderate effect variants and variants of rare frequency that could influence disease heterogeneity have been less well-characterized. Our understanding of how additional genetic variants of intermediate effect, commonly referred to as genetic modifiers, influence the severity and manifestation of causal alleles has been limited, due in part to the difficulties encountered in identifying genetic modifiers. Recent evidence also shows contribution of common variants to the risk of developing HCM (12), corroborating its genetic complexity.

Technological advances rapidly reduced the cost of genome sequencing (GS) such that application is a clinical reality. While testing continues to be focused on gene panels for primary variant identification, improved quality of sequencing chemistry and processing algorithms has enabled clinical exome and genome sequencing. GS has identified new pathogenic variants in HCM (13), as well as in diseases previously not considered genetic such as acute myocarditis (14). Data from gnomAD (15) have refined our understanding of gene tolerance to variation. We previously explored the concept of gene tolerance and its utility in classifying cardiovascular disease variants (16, 17). GS uniquely allows interrogation of all genes, regulatory regions, intergenic regions, as well as detection of larger indels and structural variants. In combination with RNA sequencing, the regulatory implications of different variants can be investigated further to improve our understanding of the genetic complexity of disease.

Here, we utilize patient derived GS and multiple sources of transcriptomic data to identify genetic modifiers of the disease phenotype in cardiomyopathy patients with a previously identified variant in *MYH7*. We develop a pipeline specific to cardiomyopathy, update curation of all putatively causal variants, and examine several aspects of variation uniquely available in genome data. In combination with transcriptional coexpression network data, we identify candidate modifiers of the cardiomyopathy phenotype. The expression profiles of several candidates are assessed in three independent models of cardiac hypertrophy.

## Methods

### Cohort

The study was approved by local IRB (GAP 4237) and conformed to the Declaration of Helsinki. We identified 48 individuals with prior genetic testing for cardiomyopathy associated variants in *MYH7*. Patients were recruited from the inherited cardiomyopathy clinics of Stanford University, University of Michigan, and the University of Chicago Medical Centers. Prior to study inclusion, all participants underwent informed consent to research-based genome sequencing. Clinical details including variant classification from the original genetic testing report were obtained. Clinical measurements and demographics are found in Tables 1, 2 and Supplementary Table S1 (echocardiography) and Data S1 (individual clinical characteristics).

**Table 1:** Baseline characteristics.

HCM						
Trait	N	Median	Mean	SD	Min	Max
Age (yrs)	42	49.5	47.2	16.1	7	79
Weight (lbs)	40	182.5	177.8	47.7	47	272
Height (inches)	40	67	66.7	5.4	48	80
Other						
Trait	N	Median	Mean	SD	Min	Max
Age (yrs)	6	46.0	39.3	21.2	8	61
Weight (lbs)	5	143	117.8	47.8	42	155
Height (inches)	5	68	61.6	10.6	44	69

**Table 2:** Demographics.

Name	Level	*N*	%
Sample origin	Site 1	26	54
	Site 2	13	27
	Site 3	9	19
Sex	F	24	50
	M	24	50
Race	Caucasian	34	71
	Hispanic	2	4
	Asian	3	6
	Middle Eastern	2	4
	African American	7	15
Morphology	Apical hypertrophy	2	4.2
	Asymmetric septal hypertrophy	30	62.5
	Burnt-out hypertrophic	1	2.1
	Concentric hypertrophy	6	12.5
	Dilated	1	2.1
	ECG+/LVH-	3	6.2
	Inferior hypertrophy	2	4.2
	Isolated noncompaction	3	6.2

### Clinical curation

Our curation process was two tiered. First, we manually curated any variant found within *MYH7* to confirm the presence of the original diagnosis and reclassified pathogenicity according to additional lines of evidence. Second, we curated a broader set of candidate myocardial genes (*n* = 452; Supplementary Table S2) to identify alternate pathogenic variants, most of which were not interrogated in the initial gene panel screening. The clinical curation protocol was based on American College of Medical Genetics (ACMG) guidelines (18, 19) as described (16). The curation classes were: (1) Very likely disease causing (equivalent to “published, disease causing mutation”; “known disease causing mutation”; “pathogenic”; “disease causing mutation”). (2) Likely disease causing (Equivalent to “presumed pathogenic mutation”; “likely pathogenic”; “probably associated”). (3) VUS, likely disease causing (equivalent to “novel variant of uncertain significance, likely disease causing”). (4) VUS (equivalent to “class II possible deleterious mutation”; “VUS”; “novel variant of uncertain significance”). (5) VUS, likely benign. Primary criteria for reclassification included population allele frequency (GnomAD greater than 0.1%), pedigree segregation, supporting publication evidence and clinical laboratory assertions available through ClinVar (March 2021).

### Sequencing, variant calling, filtering

Genome sequencing was performed on isolated genomic DNA acquired from blood samples from each participant. DNA library preparation and 2 × 100 paired-end sequencing was completed by Illumina. Coverage was on average 30× and sufficient for high quality germline variant genotyping.

Alignment and variant calling were completed using Real Time Genomics (20). Filtering was applied based on an AVR (adaptive variant rescoring) score of 0.02. Variant imputation, filtering and structural variation calling were performed using a custom pipeline as previously described (21, 22) with modification. Variants were only called at positions that had evidence for an alternate allele. Missing variant imputation was completed to avoid bias during merging samples with different sets of variants. First, the union of variants across all samples was selected. Next, each sample was interrogated for missing genotypes at these positions. Missing genotypes were set to homozygous reference if there was in excess of 10 supporting reads and no evidence of an alternative allele.

The called variants were then limited to those that were novel or had an allele frequency of less than 1% (GnomAD v2 Spring 2021). The adopted 1% minor allele frequency filter is different than what was implemented for pathogenicity as we are considering modifiers of intermediate effect and thus intermediate population frequency. Regions enriched for false positives were also removed (Undiagnosed disease program blacklist (23), ENCODE Mappability (24), Heng Li low complexity (25), GIAB low confidence (26)). To protect against overzealous filtering, variants in ClinVar (Sep 2014), GWAS NHGRI Catalog (Fall 2014) (27), PharmGKB (Fall 2014) (28), Cosmic(v70) (29) were whitelisted. ClinVar variant classifications and disease associations are current as of July 2021 (Supplementary Dataset S1).

The remaining rare high confidence variants were further limited based on functional evidence. Specifically, variants were selected that were predicted by snpEff (30) to have moderate to high impact on protein function or have a normalized CADD score (31) greater than 20. The resulting VCF was subdivided into three sets depending on the required analysis. Set 1 includes all functional candidate variants, both genic and intergenic. Set 2 includes the genic region (refFlat Fall 2014) including introns and 5 kb up and downstream of the transcription start site. Set 3 is a digital exome using a 10 bp extension of the Agilent SureSelect exome capture v2. Each of these sets was selected for burden testing in order to compare power of discovery for a gene agnostic sliding window, whole gene region and coding exome.

We used a custom pipeline to call structural variants (SVs) across the genome. As structural variant callers are known to have lower confidence particularly with short read sequences, we evaluated for SVs that were shared among multiple algorithms and restricted to no more than 20% of samples. The tools included—breakdancer v1.1.2 (32), breakseqlite v1.0, cnvnator v0.2.7 (33), delly v0.0.9 (34) and freec v1.0 (35). High confidence copy number calls are reported in the analysis. They include anything called by Breakseqlite (36) (which is considered conservative) or having greater than 50% reciprocal overlap between at least two algorithms.

### Intergenic and pathway burden testing

Intergenic regions were tested through a 50 kb sliding window with 25 kb overlap (∼5 variants). Pathways were based primarily on candidates gene sets of mitochondrial and sarcomeric function. Additionally, a large number gene sets from Reactome (37) and MsigDB (38) were used for exploratory hypothesis generation. A key challenge with burden testing is appropriate selection, tuning of the number of variants (or genes) and subsequent weighting. The default, internally calculated, frequency based weighting parameters were used with SKAT.

Aggressive post burden test filtering was applied to the case control analysis to protect against false positives due to platform bias (genome vs. exome sequencing). Genes were removed if they were previously implicated as spurious in the UDP (23) or had a prefix consistent with large paralogous gene families (OR, PRAME, MUC, POTE, BAGE, ANK, NBF, DNA, DYN, CCDC, PCD, DUX, D3X, KRT, FOX, PRSS, KIR, OPN, USP). Preliminary analysis indicated comparison of genome to exome sequencing results in spurious batch effects enriched for gene families due to capture specificity or lack thereof. Burden testing results are available in Supplementary Data S4.

### Statistical analysis

All analysis was completed using the R statistical environment and Bioconductor (39). Burden testing was performed using SKAT with adaptive weighting and adjustment for small sample sizes (40). In addition, for each region Fisher exact tests were used to compare the number of samples with rare variants among high and low groups. The high group was defined by median dichotomization for continuous traits or cases with binary trait analysis. The frequency of observed rare functional alleles was reported for each group (including a proportion test) to estimate the direction and magnitude of burden effect.

Burden testing was applied to four traits: maximum left ventricular wall thickness (LVWT), ejection fraction (EF), left ventricular outflow tract gradient (LVOTG) and case control status. The first three traits were analyzed within the cohort while case control analysis compared HCM patients against external controls (ARIC, *n* = 100) (41). For within cohort analysis a multivariate model using age, sex and BMI was used for adjustment with SKAT.

### Modifier gene prioritization

Modifier identification was performed using a gene-ranking algorithm based on a custom point system rewarding different analytical and annotation lines of evidence (Table 3). Gene-based association testing using both SKAT and SKAT-O was applied to four traits: maximum LVWT, EF, LVOTG and case control status. All gene-based association testing was restricted to unrelated Caucasian patients with clinically diagnosed HCM (*N* = 28). For within cohort analysis a multivariate model using age, sex and BMI was used for adjustment. The modifier algorithm also incorporated extensive myocardial gene expression data from heart failure and hypertrophy (42), GnomAD missense and loss of function scores, cardiac gene expression level, as well as the curated candidate myocardial gene list (Supplementary Table S2). The primary analysis was LVWT and therefore any gene reaching a nominal burden testing *p*-value < 0.05 was assigned 2 points. EF, LVOTG and Case-Control were secondary analyses and assigned 1 point each. The lowest burden test *p*-value for LVWT, EF, LVOTG and Case-Control per gene was used in the modifier scoring algorithm. Heart gene expression and significant differential expression within hypertrophy or heart failure coexpression networks (42) were each assigned 1 point. RNA expression was assessed based on the mean RPKM (reads per kilobase of exon per million reads mapped) values for 82 left ventricle GTEx (43) samples (>10 RPKM gave 1 point). GnomAD loss of function (LOF) *z*-score >3 and missense *z*-score >3 (positive z-scores indicate fewer variants than expected) each gave 1 point. Candidate genes (Supplementary Table S2), manually curated by a group of cardiologists specializing in inherited cardiovascular disease, were given 2 points. Genes previously flagged as prone to false positive signals (23) lost 2 points.

**Table 3:** Modifier gene scoring algorithm.

Trait	Criteria	Points
Left Ventricular Wall Thickness (LVWT)	Gene-based ass. test *p* < 0.05	2
Ejection Fraction (EF)	Gene-based ass test *p* < 0.05	1
Maximal Left Ventricular Outflow Tract Gradient (LVOTG)	Gene-based ass test *p* < 0.05	1
Case Control ARIC (WES, *N* = 100), (Fu et al., 2013)	Gene-based ass test *p* < 0.05	1
GTEx Gene Expression	Cardiac RPKM > 10	1
GnomAD LOF	>3 SD	1
GnomAD MIS tolerance	>3 SD	1
Hypertrophy vs. Normal (coexpression networks)	Q < 0.01	1
Heart Failure vs. Normal (coexpression networks)	Q < 0.01	1
Candidate Cardiac Gene	*N* = 452 genes	2
Flagged Prefix or UDP Blacklist		−2

### Human HCM validation

Cardiac gene expression of selected top candidate modifiers were investigated in a separate cohort of 39 HCM patients and 13 healthy control individuals (IRB approval GAP 4237). The HCM cohort (excluding one individual without available data) was 34% female and composed of individuals with a mean age of 57.9 years (standard deviation, SD = 15.7), mean height of 169.6 cm (SD = 12.8) and mean weight of 86.7 kg (SD = 34.0). RNA was isolated from cardiac left ventricle tissue from cardiac transplants or myectomies using the mirVana miRNA isolation kit (ThermoFisher Scientific) according to the manufacturer's specifications. Total RNA was reverse transcribed to cDNA using the High-Capacity cDNA Reverse Transcription kit (ThermoFisher Scientific). Quantitative real-time PCR was performed on a ViiA 7 Real-Time PCR System (ThermoFisher Scientific) using pre-designed Taqman gene expression assays from ThermoFisher Scientific (FHOD3 Hs00400902_m1, MSRB2 Hs00255292_m1, MYH7B Hs00293096_m1, PACSIN3 Hs00367625_m1, SORBS2 Hs01125197_m1) or predesigned probes from IDT (NPPB HS.PT.58.19450190). The eukaryotic elongation factor EEF1 (IDT predesigned probe Hs.PT.58.3514123) was used as a housekeeping gene, and expression was quantified using the *ΔΔ*C^T^ method followed by an unpaired *t*-test with BH-correction.

### Functional RNA-seq verification

All animal procedures were in keeping with all federal and state regulations governing the humane care and use of laboratory animals, including the USDA Animal Welfare Act, and our Assurance of Compliance with PHS Policy on Humane Care and Use of Laboratory Animals, Animal Welfare Assurance Number: A3213-01, in accordance with the NIH Guide for the Care and Use of Laboratory Animals. The laboratory animal care program at Stanford is accredited by the Association for the Assessment and Accreditation of Laboratory Animal Care (AAALAC International, Accredited Unit Number 000679). All animals were handled under protocols 22920 and 22922 approved by the Stanford Administrative Panel on Laboratory Animal Care. Mice were anesthetized with 3% isofluorane (inhalation), euthanized using cervical dislocation, after which the hearts were removed. The methods used to generate and analyze cardiac RNA sequencing data from the MYL2 transgenic mouse model have been described in detail elsewhere (44).

Neonatal rats were anesthetized on a pad on ice for 20 min and euthanized by decapitation. Neonatal rat ventricular myocytes (NRVMs) were isolated using standard methods on postpartum day 3 using the Worthington Neonatal Cardiomyocytes isolation system. After removal of non-cardiomyocyte cells, myocytes were plated on collagen I coated plates in DMEM 7.5% FBS, 7.5% horse serum, and penicillin/streptomycin. Forty-eight hours after isolation, cells were treated with 50 uM phenylephrine in low glucose DMEM with ITS and 20 mM AraC. Phenylephrine treatment (2–50uM) for 24–48 h is known to induce cardiomyocyte hypertrophy (increased cell volume, cell area and total protein content) (45–48). A subset of cells were harvested prior to addition of phenylephrine; half of remaining wells were treated with media alone. After 24 h and 48 h of treatment, acute phenylephrine induced cardiac cells were harvested. RNA was extracted using the Qiagen RNeasy kit according to the manufacturer's instructions and was DNase-treated using the DNA-free RNA kit from Zymo Research. RNA integrity was verified using a 2100 BioAnalyzer (Agilent) and all samples had an RIN score of 7.0 or higher. RNAseq libraries were prepared using the TrueSeq Stranded mRNA kit (Illumina), according to the manufacturers' instructions. Libraries were barcoded, quality-checked and run in rapid run flow cells in a HiSeq 2,500 (Illumina), producing at least 30 million paired-end reads. Sequencing reads were aligned to the Rattus Norvegicus rn5 UCSC reference genome using the STAR (49) and Cufflinks was used to quantify and perform differential expression (50). Reads were normalized using Cufflinks and FPKM (Fragments Per Kilobase Of Exon Per Million Fragments Mapped) was calculated on a per gene basis. We evaluated the FPKM count for top candidate modifiers and canonical markers of the acute hypertrophic program and evaluated the differential expression after phenylephrine vs. pre-treatment as a ratio vs. sham vs. pretreatment. All genes with an FPKM>1 were considered expressed and included in the analysis.

## Results

### Genome sequencing of patients harboring *MYH7* variants

**Figure 1:**
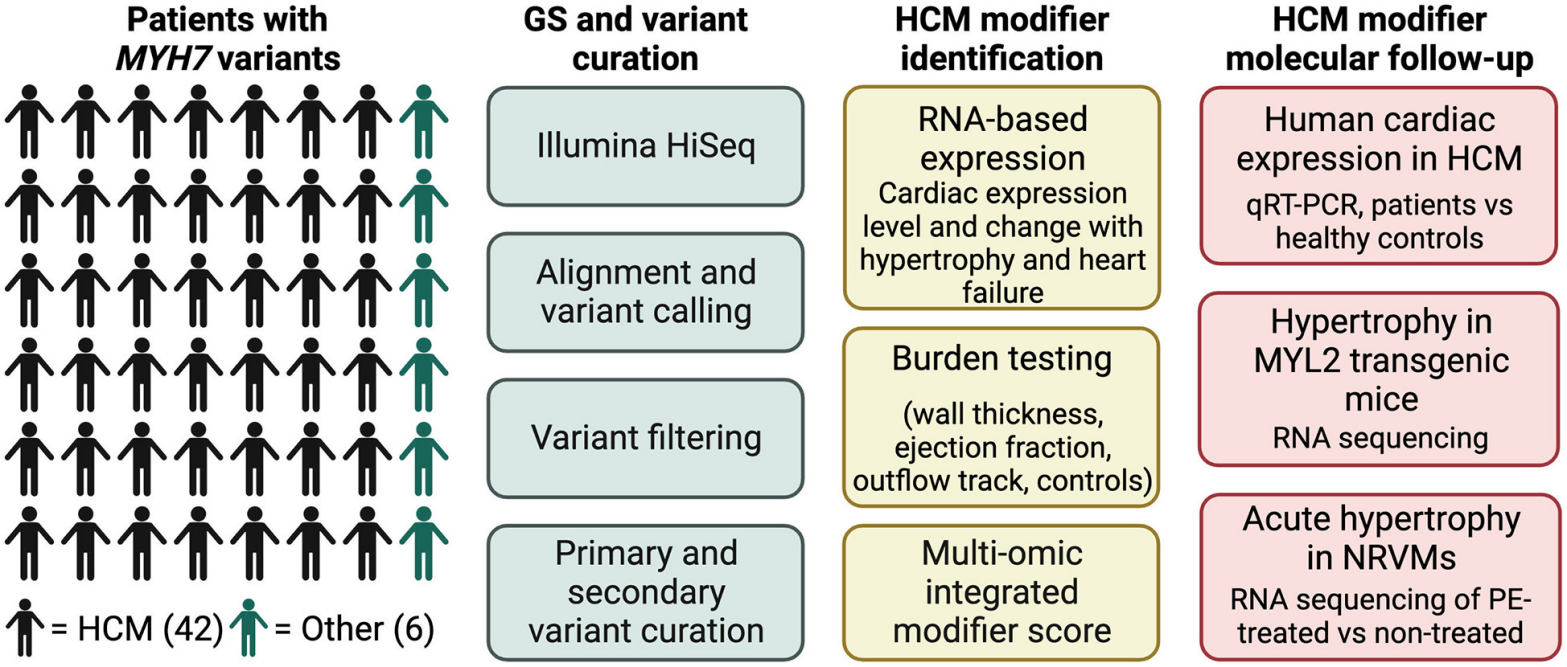
Overall study design and workflow. Main patient cohort (*N* = 48) all harbored *MYH7* variants. 42 patients had been diagnosed with HCM, and 6 had other diagnoses. Genome sequencing (GS) was performed with an Illumina HiSeq on all patients, followed by variant calling and curation. HCM candidate modifiers were identified through a scoring system that utilized a combination of RNA expression data, gene-based association testing and previous candidate hypertrophy-associated genes. Molecular follow-up of top modifier candidates was performed in three separate models of cardiac hypertrophy.

Patients were classified based on cardiac morphology and *MYH7* variant identified (primary variants identified between 2007 and 2011, *n* = 33, Tables 1-2, Supplementary Table S1 and Supplementary Data S1). Of the 48 patients (7 to 79 years old), 50% were female. The cohort included 6 families for a total of 40 unrelated individuals. Cardiomyopathy phenotypes were classified as hypertrophic in 42, dilated without known prior hypertrophy in 1, and noncompaction in 3. The overall study design is illustrated in Figure 1.

**Figure 2:**
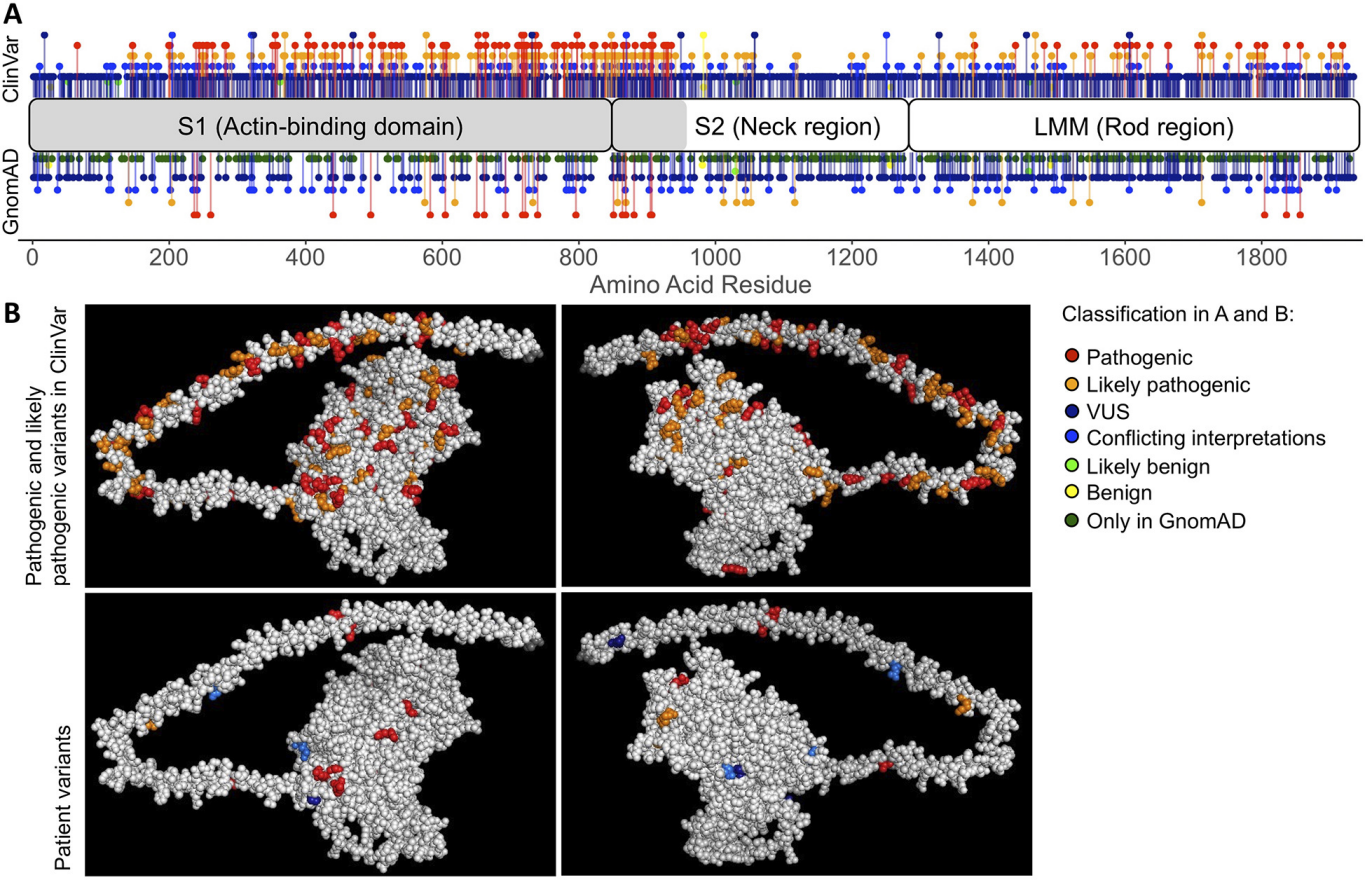
Distribution of *MYH7* variants. **(A)** Variants observed in ClinVar and GnomAD across the length of MYH7, 1935 amino acid residues in total. The gray region of *MYH7* (residues 1-959) is illustrated in the 3D model in **(B)**. **(B)** Top panels (different sides of the protein) show all variants classified as pathogenic or likely pathogenic in ClinVar, including patient variants classified as pathogenic or likely pathogenic. At amino acid residues where multiple variants of different classification are present, the residue is colored based on the variant with maximum severity. Residues in both A and B are colored based on ClinVar classification with pathogenic variants in red, likely pathogenic variants in orange, variants with conflicting interpretations of pathogenicity in blue, variants of uncertain significance (VUS) in dark blue, likely benign variants in green and benign variants in yellow. GnomAD variants without a ClinVar classification are shown in dark green.

Multiple genetic variants of different classifications in *MYH7* have been reported in GnomAD (15) and ClinVar (51) (Figure 2A). Using our automated genome annotation pipeline, we re-identified all unique *MYH7* variants detected on initial panel-based sequencing (population minor allele frequency <0.1%, Figure 2B bottom panels, Supplementary Dataset S1). They were distributed across the actin-binding domain and the rod region, similar to the majority of reported variants in ClinVar (Figure 2B, top panels). All *MYH7* variants reported on prior clinical testing were identified and called at high quality and adequate depth to confidently call heterozygosity. All other genomic variants identified on panel testing were confirmed.

### *MYH7* variant reclassification

#### Secondary findings—coding

We next searched for secondary likely pathogenic genetic variants that may contribute to the cardiomyopathy phenotype (Supplementary Data S2). In addition to potentially disease-associated variants in *MYH7* from panel based testing, we identified one *MYBPC3* VUS (p.Val757Met), and rare *PRKAG2* variants (p.Val535Gly and p.Val534Gly respectively). We identified an early termination (p. Cys1013X) and rare missense (p.Lys953Gln) variant in *FLNC*, a gene first implicated in hypertrophic and dilated cardiomyopathy (DCM) years after the patients' clinical tests were reported (52, 53). Interestingly, the former *FLNC* truncation variant was found in a patient with a family history of DCM whose *MYH7* variant was reclassified to benign. Another patient whose *MYH7* variant was initially classified as VUS also had a rare nonsynonymous variant in RNA binding motif protein 20 (*RBM20* p.Ile921Val), a titin-splicing gene implicated in cardiomyopathy (54). Two rare variants in BCL2-associated athanogene-3 (*BAG3* p.Glu471Gly and p.Arg473Gly), a stress responsive protein implicated in familial DCM were also identified (55–57). The *MYH7* Variant Reclassification is summarized in Supplementary Figure S1.

Review of rare coding variants in a cardiovascular subset of the 78 ACMG gene list (58) identified additional rare variants (Supplementary Data S2). These were found in genes associated with arrhythmogenic right ventricular cardiomyopathy (*DSC2*, *DSG2*, *TMEM43*, *PKP2*, *DSP*); Catecholaminergic polymorphic ventricular tachycardia (*RYR2*, *CASQ2*, *TRDN*); DCM (*LMNA*, *FLNC*, *TTN*, *BAG3*, *RBM20*); Vascular Ehlers-Danlos Syndrome (*COL3A1*); Long QT Syndrome 3/Brugada Syndrome (*SCN5A*); Familial Hypercholesterolemia (*LDLR*, *APOB*) and aortopathies (*TGFBR1*, *SMAD3*, *MYH11*).

#### Secondary findings—non-coding

Next, we interrogated miRNA binding sites and consensus promoter regions for potentially interesting variants (Supplementary Data S3). We identified two variants in conserved miRNA binding sites of genes previously implicated in cardiomyopathy: *SCN5A* (chr3:38589677) and *RAF1* (chr3:12625903)*.* The *RAF1* miRNA binding site variant alters position 5 of a consensus seed recognition sequence. This variant is predicted to disrupt a permissive consensus binding site recognized by multiple miRNAs and found in more than 290 genes. We also identified a variant in a miRNA binding site of *ACTN2* (chr1:236927207), a gene for which we and others recently demonstrated that protein-truncating variants cause hypertrophy and restrictive cardiomyopathy (RCM) in humans (59). We found variants of uncertain significance in non-coding RNA, promoter, UTR, enhancers and nonsynonymous variants in alternative isoforms of tropomyosin found in long-read heart RNAseq data (Supplementary Table S3). Published work demonstrates a protective role of long non coding RNAs (*mhrt)* that reside in the 3' myosin tail-coding domain and downstream of *MYH7* (60). We identified 5 rare nonsynonymous variants that intersect with exons of these transcripts (Supplementary Data S3 and Table S3). As these variants affect both coding and non-coding transcripts, the impact of these variants is currently unknown. Reclassification (61) of these variants without knowledge of the role of their impact on *Mhrt* transcripts resulted in downgraded pathogenicity.

#### Scoring algorithm for modifier identification

We developed a multi-omic cardiomyopathy-data rich metric of modifying potential to evaluate for genetic modifiers of the HCM phenotype. To prioritize genes, we developed a 12-point scale (Table 3; Methods) that combined rare variant gene-based association testing with multiple external priors, including extensive myocardial gene expression data from heart failure and hypertrophy. Inclusion of parameters such as LVWT and disease-specific gene expression data enhanced sensitivity to an HCM-specific phenotype and cardiac function. There were 165 genes that reached a modifier score of 6 or better (Supplementary Table S4, see Supplementary Data S4 for full table) and 51 genes had scores ≥7. *CACNA1C* scored 10, while *RYR2*, *TTN, ATP1A2*, *FHOD3*, *TJP2, CACNA1D* and *DYNC1H1* had modifier scores of 9. Several were identified as *a priori* candidates. The sodium-potassium ATPase subunit alpha 2 gene (*ATP1A2*) was highly significant in the case-control comparison (*p* < 4 × 10^−19^). The cardiac specific ryanodine receptor encoded by *RYR2* was associated with increased wall thickness (*p* < 0.01), a trend towards LVOT gradient (*p* = 0.07) and with HCM in the case-control comparison (*p* < 0.05). Several variants in the L-type calcium channel component *CACNA1C* have also been demonstrated to predispose for arrhythmic phenotypes, especially long-QT syndromes (62), while the gap junction protein encoded by *TJP2* has been associated with hypertrophy in other cell types (63).

Validating our approach, the known modifier Formin homology 2 domain containing 3 (*FHOD3*) was a top scoring modifier. *FHOD3* has been implicated as disease predisposing in DCM and HCM (64, 65) and as an HCM modifier in case-control common variant analysis and a genome-wide association study (12, 66). Previously cited high frequency variants (rs516514, rs2303510) were not included in the rare functional variants used for gene-based association testing; despite this, variation in *FHOD3* was associated with increased LVWT (*p* < 0.05). *SORBS2* (sorbin and SH3 domain-containing 2), modifier score 6, is an adhesion junction protein that was recently associated with arrhythmogenic cardiomyopathy (ACM). Knockdown of *SORBS2* in mice resulted in an ACM-like phenotype and two patients with ACM have been identified to carry likely pathogenic variants in *SORBS2* (67).

We identified several genes as novel candidate modifiers of the HCM phenotype (Supplementary Table S4). Rare variant gene-based association testing of *DYNC1H1* (score 9), the heavy chain component of dynein, was associated with increased LVWT (*p* < 0.01) and reduced EF (*p* < 0.01). Another top modifier, *HNRNPC* (score 8), was recently identified as a regulator of sarcomeric protein translation with higher expression in failing hearts (68). *ERBB2* (score 8) and *ERBB4* (score 7), both Neuregulin-1 receptors, are involved in cardiac regeneration after injury (69, 70), and Neuregulin-1 itself is a therapeutic target for heart failure (71). Rare variants in *CAMSAP1* (score 7) were found in 8 participants with severely increased LVWT but not in participants with milder hypertrophy (*p* < 0.001). *ASH1l* (score 7), a histone H3 methyltransferase, was most significant in the case-control comparison (*p* < 1 × 10^−35^). The transcription cofactor LIM domain binding 2 (*LDB2*, score 6) was associated with LVWT (*p* < 0.01) and with case status in case-control analysis (*p* < 2 × 10^−15^). *PACSIN3* (score 6) is an adapter protein that regulates membrane dynamics. Cardiomyocytes in *PACSIN3* knockout mice lack caveolae (72), while its potential modifying function in cardiac hypertrophy remains to be elucidated.

### Independent experimental RNA profiling of identified candidate modifiers

**Figure 3:**
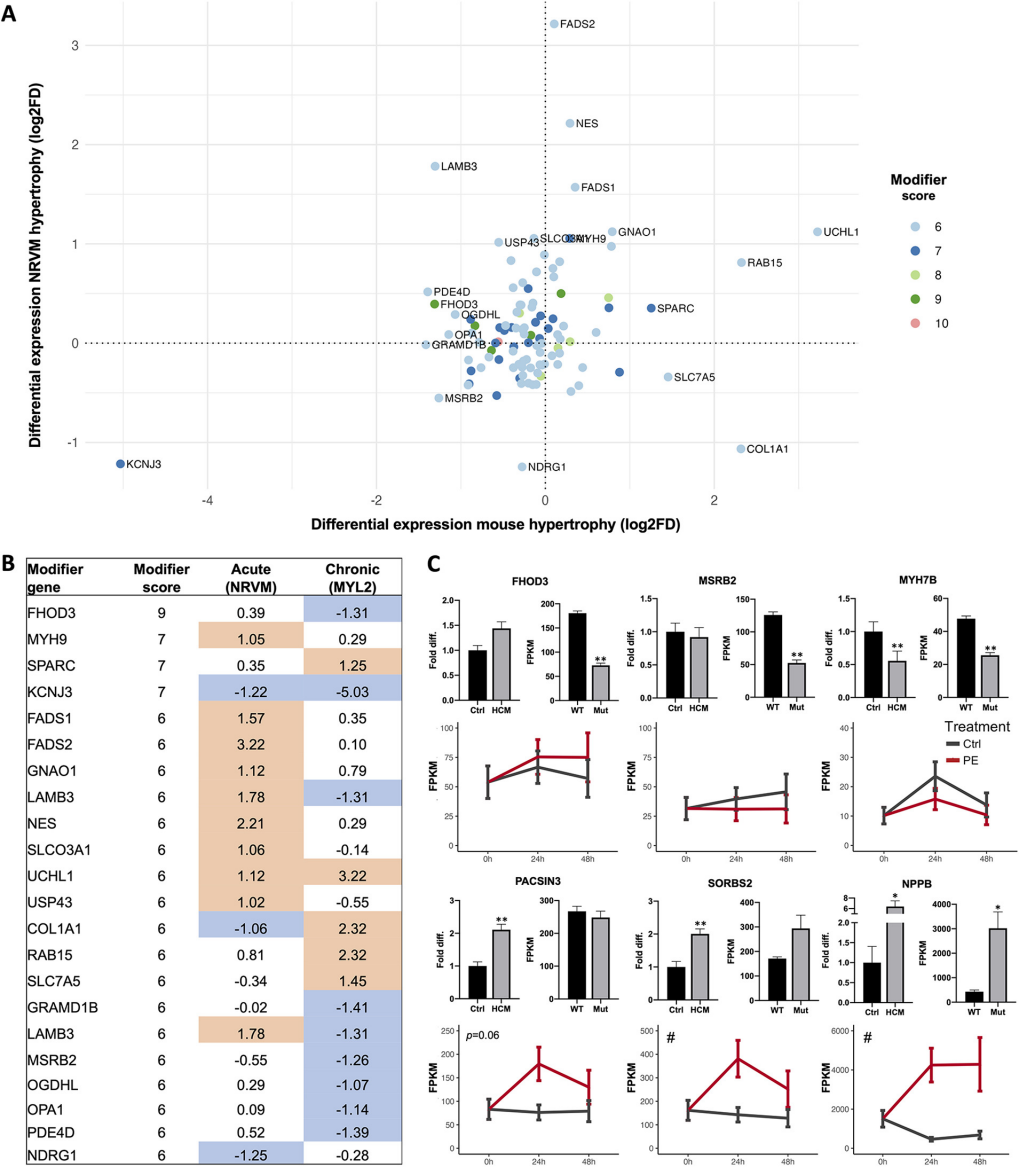
Cardiac candidate modifier gene expression changes. **(A)** Differential expression analysis of all candidate modifiers with a score 6 or higher that were expressed in both a chronic model of hypertrophy (*MYL2* transgenic mice compared to WT mice, *x*-axis) and an acute model of hypertrophy [phenylephrine-treated (PE) compared to non-treated (Ctrl) neonatal rat ventricular myocytes (*N*RVMs), *y*-axis]. Expression changes are shown as log2 of the fold difference and genes are colored based on modifier score. **(B)** Table of all labeled candidate modifiers in **(A)**, with score and log2FD listed for the acute and chronic hypertrophy models. Blue indicates negative change (log2FD -1 or lower), orange indicates positive change (log2FD 1 or more). **(C)** Differential expression of selected modifiers and the hypertrophy marker *NPPB* was investigated in a separate cohort of human HCM compared to healthy control left ventricle cardiac tissue, shown in the top left panel for each gene. Differential expression is also reported for the chronic hypertrophy model (*MYL2* transgenic mice compared to WT, top right panel for each gene) and in the acute model of cardiomyocyte hypertrophy (PE, red lines) of NRVMs. Candidate modifier expression in human cardiac left ventricle samples was investigated with qRT-PCR (*N* = 34-39 HCM patients for all factors, and *N* = 5-13 control individuals for each factor). Expression in *MYL2* transgenic mice (Mut, *N* = 3 mice) vs. littermate WT controls (WT, *N* = 3 mice), and in PE treated (*N* = 3 wells per time point) and non-treated (*N* = 3 wells per time point) NRVMs was investigated using RNA sequencing. For NRVMs, results are shown for 3 time points: 0 h when PE was added, after 24 h of PE treatment and after 48 h of PE treatment. NRVM data is shown as mean ± 95% CI (# indicates *p* < 0.05 ANOVA treatment effect), human and mouse data as mean ± SEM, * *p* < 0.05, ** *p* < 0.01 (BH-corrected unpaired *t*-test).

To further evaluate if the identified candidate modifier genes were involved in cardiac hypertrophy, we analyzed RNA sequencing data from a mouse model of RCM and a cellular model of acute hypertrophy in neonatal rat ventricular myocytes (NRVMs). We specifically investigated the relative change in expression for all modifier genes with a score of 6 or above (*n* = 165) in response to the chronic and acute hypertrophic stimulus (*n* = 106 detected in both, Figures 3A,B, Supplementary Table S5, with low-scoring genes shown in Supplementary Figure S2A). While a change in expression suggests a role in development of cardiac hypertrophy, lack of change does not invalidate a potential modifier. We confirmed several genes as highly potentiated by a hypertrophic signal, including the known modifier *FHOD3*, and *UCHL1* (Supplementary Figure S2B), for which knockdown in cardiomyocytes was recently shown to prevent hypertrophy (73). In contrast, other modifiers were modestly changed or expressed at low levels in cardiomyocytes, such as *CAMSAP1* and *ASH1l*. The most downregulated modifier in response to both acute and long-term hypertrophy was the potassium channel gene *KCNJ3* (score 7, Supplementary Figure S2C). Genetic variation in *KCNJ3* is associated with arrhythmias, and blocking the channel in zebrafish improved the arrhythmogenic phenotype (74).

Next, we analyzed gene expression differences in human left ventricular myocardial tissue from an independent cohort of 39 HCM patients compared to healthy controls (*N* = 13). Expression of the hypertrophy marker *NPPB* and five candidate modifiers (*FHOD3, MSRB2, MYH7B, PACSIN3* and *SORBS2*) in all three experiments are shown in Figure 3C. The expression changes in *SORBS2* follows the effect of *NPPB*, suggesting a role in hypertrophic remodeling. To further explore the utility of gene expression data in identifying and explaining the roles of modifiers, we assessed the expression of genes with recently-identified HCM susceptibility loci (8, 12) in our cardiomyopathy models. Genes with known cardiac functions, including *BAG3*, *PLN*, *TBX3*, *TRDN*, and *MTSS1*, all had lower expression in both the acute and chronic cardiomyopathy compared to controls (Supplementary Figure S3). *SLC6A6*, a taurine transporter, was higher in both models. *ADPRHL1*, a gene implicated in cardiac development, was upregulated in response to acute hypertrophy and downregulated in the chronic hypertrophy model (Supplementary Figure S3, Table S5).

## Discussion

Genome sequencing offers unique and untapped power for understanding the complexity of genetic disease. Variability in severity, penetrance, and phenotype found within and among families harboring putatively disease-causative *MYH7* variants is presumed to be caused by both genetic and environmental modifiers. We evaluated the utility of GS in combination with cardiac expression data to identify candidate genetic modifiers in a patient cohort initially diagnosed with monogenic cardiomyopathy attributed to variants in *MYH7*.

Whereas prior studies of GS in HCM have focused on its utility in providing a genetic diagnosis in cardiomyopathy (13, 75), we showed how comprehensive data from GS of genetically diagnosed HCM patients identified potential genetic modifiers of the phenotype. Increasing evidence suggests that HCM is not a wholly monogenic disease. We searched each genome for evidence of additional rare variants causative of or substantially contributory to the observed cardiomyopathy phenotype and found rare protein altering variants in multiple genes and individuals, including an early termination variant in the cardiomyopathy associated gene *FLNC*. A VUS in *FLNC* in the presence of a VUS in *MYBPC3* has previously been suggested as a potential modifier of HCM (75). These findings support previously identified advantages of GS in HCM, including the potential to improve yield in testing for primary pathogenic variants (13), and identify a range of secondary findings throughout the genome (75). Further exploration of secondary genetic variants, where gene expression is one potential tool, is necessary to validate their significance in disease pathophysiology and translate such findings into clinically actionable insight.

Novel potential genetic contributors to disease were discovered in the non-coding genome, including microRNA sites and lncRNAs. The discovery of non-coding DNA variants that may act as disease modifiers is the first step in developing a new field of biology aimed at understanding how regulatory gene variants impact Mendelian disease; this is fodder for applied genetic research. Further mechanistic research is needed to understand the impact of non-coding variants in the genetic basis for HCM.

We further explored the genomes for modifiers of disease phenotype. With limited sample size to afford true statistical power for genome wide testing we undertook two strategies to maximize the robustness of findings. We used a gene scoring algorithm that boosted nominally significant modifier phenotype associations with specialized annotation of hypertrophic cardiomyopathy and heart failure molecular phenotypes. We prioritized variants based on cardiac expression and differential expression in cardiac hypertrophy and heart failure. Several candidates were investigated for cardiac expression changes in a separate cohort of human HCM, a transgenic mouse model of chronic hypertrophy and an acute hypertrophy model in NRVMs. Our top candidates included previously known modifier genes (*FHOD3*), a gene known to cause DCM and HCM when mutated (*TTN*) and genes associated with related cardiovascular phenotypes such as heart failure (*HNRNPC)* and arrhythmia (*CACNA1C*, *RYR2*). The plausibility, and in the case of for example *FHOD3*, *SORBS2* and *UCHL1*, replication of prior published results, lends credibility to our method and strengthen the discovery of novel candidates such as *CAMSAP1*, *MYH7B* and *PACSIN3.* Importantly, rare genetic variation does not inform directionality of effects, and utilizing several disease models for investigation of potential modifier expression changes in response to acute or chronic hypertrophy emphasizes the complexity of the observed effects. For *FHOD3*, for example, expression was substantially lower in RCM mice, but showed a trend towards higher levels in the HCM validation cohort and in response to acute hypertrophy.

Our study has several limitations. Our sample size is limited and includes mostly Caucasian individuals. The modifier scoring algorithm was developed to include cardiomyopathy-relevant metrics, but is limited by its scope and selection of scoring cutoff. While we further evaluated some of the modifiers using cardiac hypertrophy models and a separate HCM cohort, larger cohort studies are needed to examine their potential clinical relevance and future mechanistic studies to establish causal evidence. Importantly, the cell and animal models are limited with regards to cross-species concordance and the potential impact by acute or chronic hypertrophy at two time points. They are therefore unlikely to capture all differential signaling that influence human HCM development over time. Lack of validation thus does not invalidate a putative modifier. In addition, identified phenotypic associations were concurrent with the point of assessment. Hence, the phenotypic association is contingent upon the gene's direct impact but also on potential gene-mediated influences on ascertainment timing, treatment history etc. The test statistics and *p*-values will be inflated/deflated due to multiple tests using sparse data and should therefore only be used for ranking purposes and not to determine actual statistical significance. Furthermore, no validation was performed at the protein level, which will be an important avenue for future research as proteomic and phosphoproteomic signatures also impact disease severity in sarcomeric HCM (76, 77).

In summary, our results provide new insights into genetic modifiers of HCM, including disease-associated genes, proximal regulatory regions and non-coding variants. The complexity of secondary genetic variants with respect to modifying effects is currently minimally evaluated. The initial cataloging of these gene-gene interactions are preliminary and will require refinement and extension, just as the initial discovery of causal *MYH7* variants have been. We hypothesize that these secondary findings provide greater understanding in the interpretation of primary variants, and could eventually provide utility in predicting disease severity. True modifiers of disease severity represent promising targets for drug therapy irrespective of causative variants and warrant further mechanistic investigation.

## Data Availability

The genome data presented in the current publication have been deposited in and are available from the dbGaP database under dbGaP Study Accession phs004024.v1.p1.
