# Supplementary material for: Identification of candidate cardiomyopathy modifier genes through genome sequencing and RNA profiling
Source: Front Cardiovasc Med. 2025 Jul 28;12:1546493. doi: 10.3389/fcvm.2025.1546493 (PMC12336239; doi:10.3389/fcvm.2025.1546493)
Supplement: Supplementary file 1 [file Presentation1.pdf]

SUPPLEMENTAL MATERIAL

Supplemental Figures and Legends

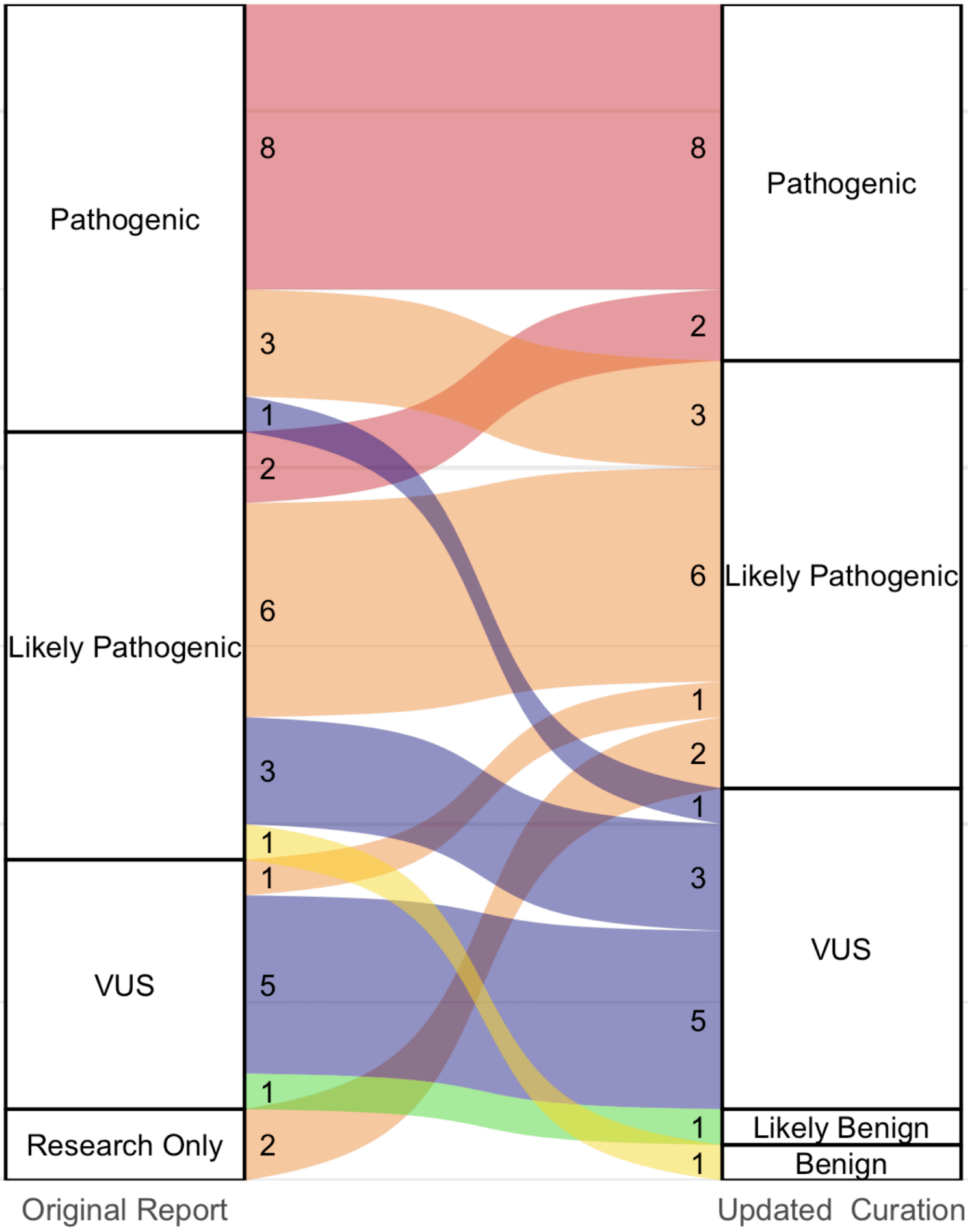

Figure S1. MYH7 variant reclassification. Depicts changes in *MYH7* variant classification from time of original report (left) to publication (right).

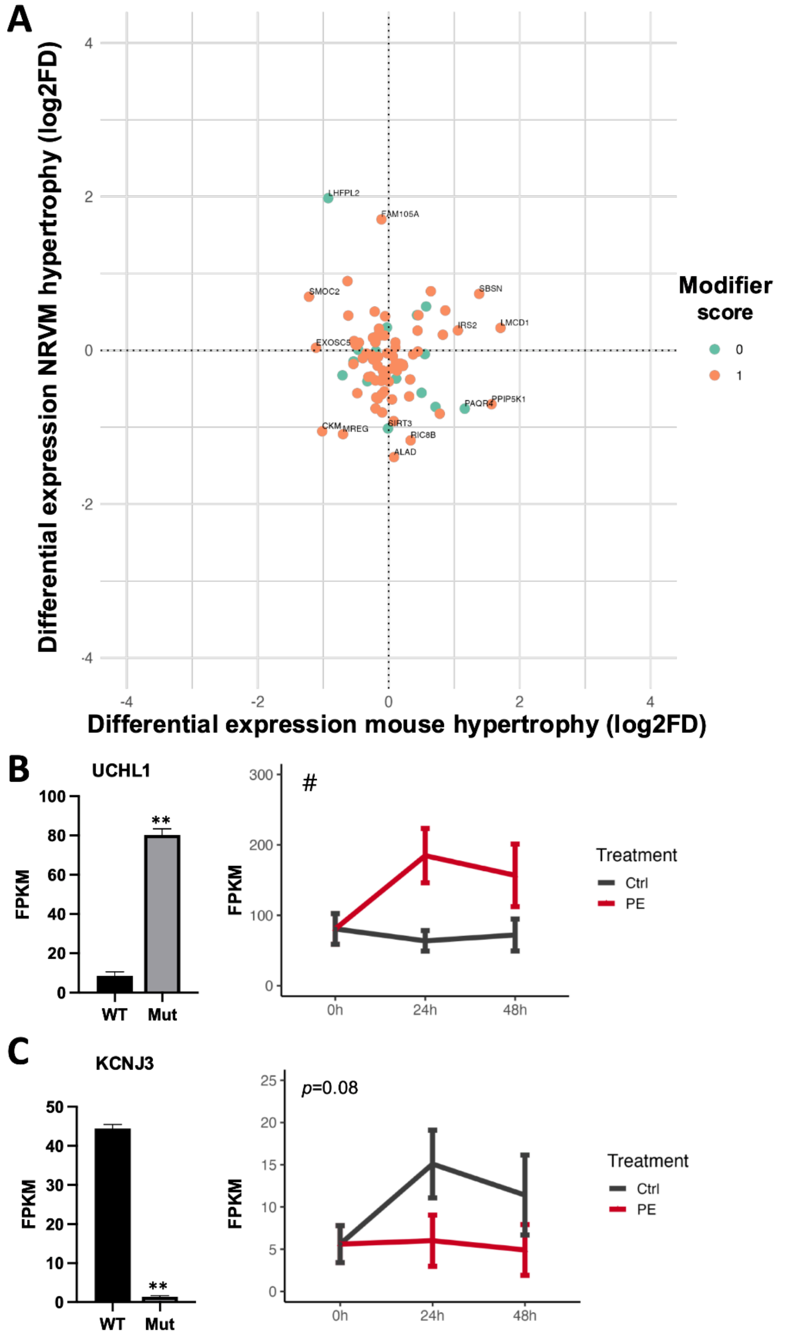

Figure S2. A) Differential expression analysis of all candidate modifiers with a score 1 or lower that were expressed in both a chronic model of hypertrophy (*MYL2* transgenic mice compared to WT mice, x-axis) and an acute model of hypertrophy (phenylephrine-treated (PE) compared to non-treated (Ctrl) neonatal rat ventricular myocytes (NRVMs), y-axis). Expression changes are shown as log2 of the fold difference and genes are colored based on modifier score. B-C) Effects of long-term (left) and acute (right) rodent models of hypertrophy on gene expression of B) UCHL1 and C) KCNJ3. Long-term: Expression in *MYL2* transgenic mice (Mut, N=3 mice)

versus littermate WT controls (WT, N=3 mice). Acute: PE treated (N=3 wells per time point) and non-treated (N=3 wells per time point) NRVMs, investigated using RNA sequencing. For NRVMs, results are shown for 3 time points: 48 hours after isolation when PE was added, 72 hours after isolation (after 24 hours of PE treatment) and at 96 hours after isolation (after 48 hours of PE treatment). NRVM data is shown as mean ± 95% CI (# indicates $p<0.05$ ANOVA treatment effect), and mouse data is hown as mean ± SEM, * $p<0.05$, ** $p<0.01$ (BH-corrected unpaired t-test).

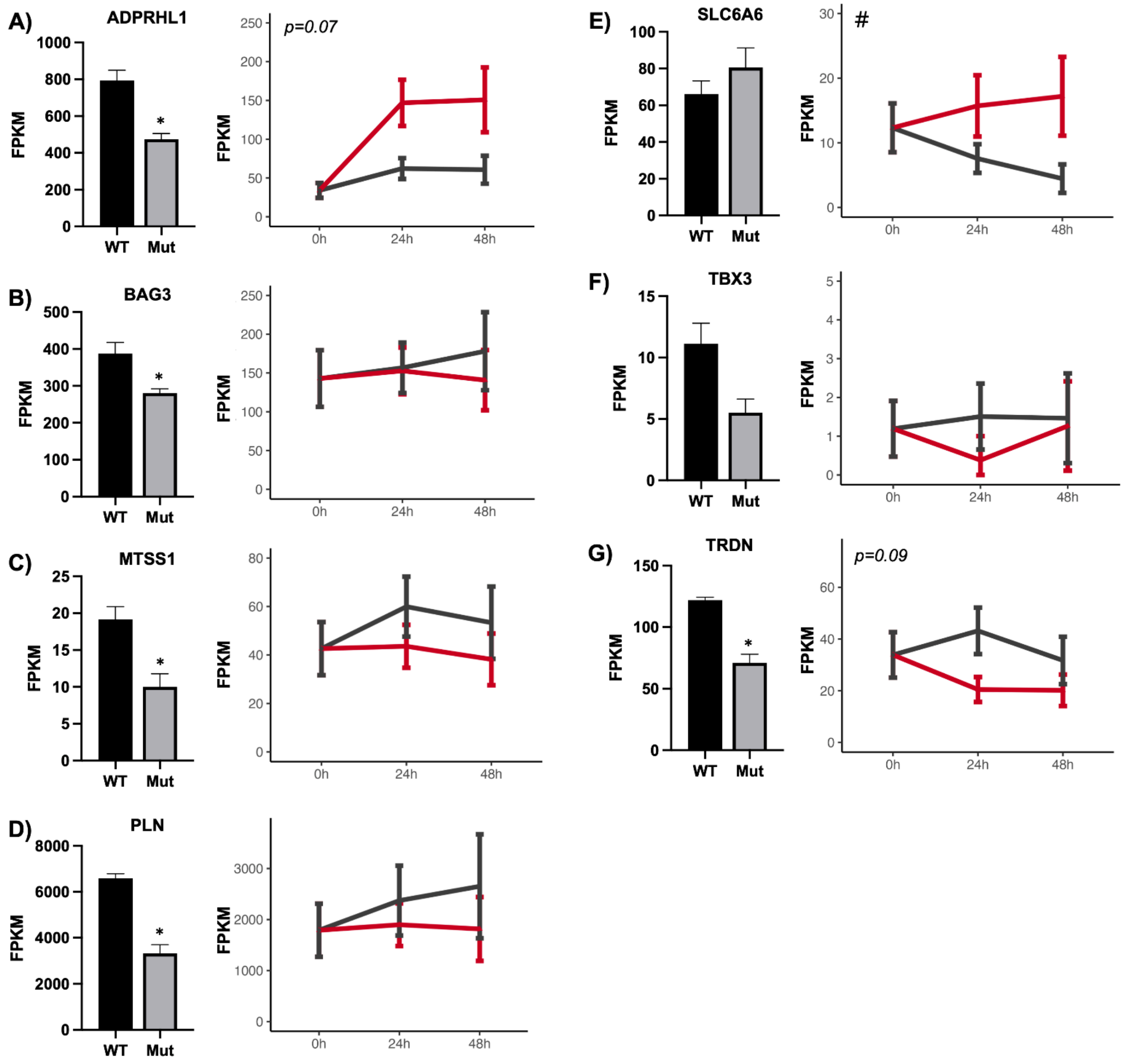

Figure S3. Expression of genes with identified HCM susceptibility loci from [31, 32]. Effects of long-term (left) and acute (right) rodent models of hypertrophy on gene expression of A) ADPRHL1, B) BAG3, C) MTSS1, D) PLN, E) SLC6A6, F) TBX3 and G) TDRN. Long-term: Expression in *MYL2* transgenic mice (Mut, N=3 mice) versus littermate WT controls (WT, N=3 mice). Acute: PE treated (N=3 wells per time point) and non-treated (N=3 wells per time point) NRVMs, investigated using RNA sequencing. For NRVMs, results are shown for 3 time points: 48 hours after isolation when PE was added, 72 hours after isolation (after 24 hours of PE treatment) and at 96 hours after isolation (after 48 hours of PE treatment). NRVM data is shown as mean ± 95% CI (# indicates $p<0.05$ ANOVA treatment effect), and mouse data as mean ± SEM, * $p<0.05$ (BH-corrected unpaired t-test).

Supplemental Tables

Table S1. Echocardiography parameters.

Table S2. Candidate cardiac genes.

Table S3. Secondary findings within non-coding regions.

Table S4. Top Ranked Modifier Genes by Modifier Score.

Table S5. Fold differences of modifiers in experimental models of acute and long-term hypertrophy (phenylephrine-treated vs non-treated NRVMs, and MYL2 transgenic vs wild-type mice).

Supplemental Datasets

Supplemental Data S1. Clinical characteristics and *MYH7* Variant Curation.

Supplemental Data S2. Rare variants identified with GS (maximum allele frequency of 1%, protein altering coding regions)

Supplemental Data S3. Candidate variants in miRNA binding sites.

Supplemental Data S4. Full modifier dataset including gene-based association testing results.
